# Hyperthermia-Induced Changes in EEG of Anesthetized Mice Subjected to Passive Heat Exposure

**DOI:** 10.3389/fnsys.2021.709337

**Published:** 2021-09-09

**Authors:** Carmen de Labra, Jose L. Pardo-Vazquez, Javier Cudeiro, Casto Rivadulla

**Affiliations:** ^1^NEUROcom, Departamento de Fisioterapia, Medicina y Ciencias Biomédicas, Facultade de Ciencias da Saúde, Universidade da Coruña (UDC), A Coruña, Spain; ^2^Centro de Investigaciones Científicas Avanzadas (CICA), Universidade da Coruña (UDC), A Coruña, Spain; ^3^Instituto de Investigación Biomédica de A Coruña (INIBIC), Complexo Hospitalario Universitario de A Coruña (CHUAC), A Coruña, Spain; ^4^Centro de Estimulación Cerebral de Galicia, A Coruña, Spain

**Keywords:** hyperthermia, electroencephalography, physiology, mice, anesthetized animal

## Abstract

Currently, the role of hypothermia in electroencephalography (EEG) is well-established. However, few studies have investigated the effect of hyperthermia on EEG, an important physiological parameter governing brain function. The aim of this work was to determine how neuronal activity in anesthetized mice is affected when the temperature rises above the physiological threshold mandatory to maintain the normal body functions. In this study, a temperature-elevation protocol, from 37 to 42°C, was applied to four female mice of 2–3 months old while EEG was recorded simultaneously. We found that hyperthermia reduces EEG amplitude by 4.36% when rising from 37 to 38 degrees and by 24.33% when it is increased to 42 degrees. Likewise, increasing the body temperature produces a very large impact on the EEG spectral parameters, reducing the frequency power at the delta, theta, alpha, and beta bands. Our results show that hyperthermia has a global effect on the EEG, being able to change the electrical activity of the brain.

## Introduction

Mammals keep their body temperature within certain boundaries, as it is the base of most biological processes. Considering the electrophysiological responses in the nervous system, changes during profound hypothermia have been described, consisting of a flat line or an isoelectric electroencephalography (EEG), with a total recovery once the temperature returns to normal (Gaenshirt et al., 1954; Pearcy and Virtue, 1959). While loss of EEG activity during hypothermia is widely accepted, data about the effect of hyperthermia on nervous system activity are less clear, as different brain areas have been studied, various exposure times and heating techniques have been used, and diverse animal species have been explored (Sminia et al., 1994). Using rats and a thermostatically controlled chamber, Gold et al. (1985) found no significant correlation between changes in body temperature and EEG. Lifshitz et al. (1987), in patients with fever and without neurologic disease, found that 6 of the 14 subjects had completely normal EEG activity. Additionally, in humans, but with a protocol of deliberate hyperthermia, Dubois et al. (1980, 1981) found a diffuse decrease in cortical activity that varied with the extent and duration of hyperthermia and was reversible after a few hours of cooling. With a similar protocol, Reilly et al. (1980) demonstrated a heat-induced decline in both the frequency and amplitude of the EEG.

In recent years, there has been renewed interest in EEG recordings, which is largely due to both increased real-time EEG monitoring of brain function in critical patients, such as coma or epileptic patients (Crepeau et al., 2015; Faust et al., 2015; Kubota et al., 2018) and because neuroimaging techniques are not always available because of their elevated cost. In this scenario, and for many researchers, EEG is the preferred technique for data acquisition of cerebral activity (Fingelkurts et al., 2013), in humans and in experimental models.

In this sense, the mouse is becoming the reference animal in brain research; mice are affordable, require limited facilities, and transgenic methodology can be used to create mouse models of human diseases. As genetic manipulations became more sophisticated, neuroscientists increased the availability of mouse models to work with. Particularly in epilepsy, there are several models where temperature is a critical factor for triggering the crisis (van Gassen et al., 2008; Oakley et al., 2009; Ricobaraza et al., 2019), however, to the best of our knowledge, there are no studies examining the changes in mouse EEG during hyperthermia. Because of this factor, and because elevated temperature may induce severe damage and complications in the nervous system, the aim of this investigation was to study the effect of whole-body hyperthermia on the brain activity of mice.

## Materials and Methods

All the methods were carried out in compliance with the ARRIVE (Animal Research: Reporting of *in vivo* Experiments) guidelines. This study was conducted according to the rules of Physiological Spanish Society, International Council for Laboratory Animal Science, and the European Union guidelines (No. 2010/63/EU) for the protection of laboratory animals used for scientific purposes. The experimental protocol was approved by the ethics committee for animal research of the University Hospital of A Coruña.

Four female mice, 2- to 3-month-old C57-BalbC mice, were prepared and used for the experiments. Mice were maintained under standard laboratory conditions on a 12/12-h light/dark cycle with free access to food and water.

### Surgery

For surgery, animals were anesthetized with sevoflurane (3.5% for induction and 2–2.5% for maintenance) and positioned on a heating pad, with a rectal probe inserted, to maintain their core temperature at 37°C (Thermostatic blanket low noise, model RTC-1, Cibertec, Madrid, Spain). Once the animal was stabilized, it was placed in a custom-made frame, the skin was open, and the connective tissue was removed until the lambda and bregma landmarks were visible. Using a high-speed dental drill, two small craniotomies were made in each mouse over the right somatosensory (AP −1.5 mm from the bregma, L 2.5 mm from the midline) and left primary visual (AP −2.5 mm, L 1.5 mm) cortices. At these locations, two copper wire electrodes (length = 2 cm, diameter = 0.2 mm) were implanted with their tips touching the dura for EEG recordings. Electrodes were fixed with a drop of fast drying superglue and a thin layer of dental acrylic. The electrode located on the somatosensory cortex was the active electrode, and the other electrode was the reference electrode.

### Data Acquisition

Recordings started 1 day after the surgery. The EEG signal was bandpass filtered (gain 1,000, range 1–500 Hz), amplified using an A-M system differential amplifier (Model 1700 A-M System, LLC, Calsborg, WA, United States), digitized at 20 kHz (1401 CED A/D convertor card; Cambridge Electronic Design, United Kingdom) and stored (Spike 2 software; Cambridge Electronic Design, United Kingdom) in a PC for online checking and posterior analysis.

### Study Design

**FIGURE 1:**
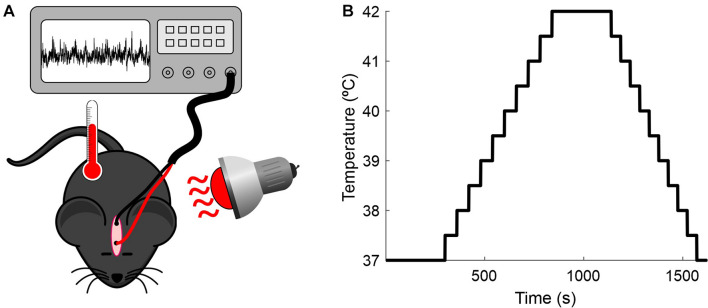
Recording setup. **(A)** Experimental preparation. Anesthetized mice were placed under a heat lamp while the core temperature was monitored and the EEG recorded. **(B)** Protocol for increasing the mice’s temperature.

The experimental setup and the temperature-elevation protocol used in this study are illustrated in Figure 1. The measurements were conducted for up to 6 days in each animal for a total of 19 sessions. Each day the animal was anesthetized as described above, two alligator clips were clamped to the implanted electrodes, and the rectal temperature probe was placed and connected to the mouse temperature controller (RTC-1 System, Cibertec, Spain). The body temperature was initially maintained for 5 min at 37°C by a heat lamp positioned near the mouse (baseline). After this period, body temperature was continuously increased (0.5°C/min) from 37 to 42°C, where it was maintained for 5 min. Following this step, the mouse was cooled until the core temperature reached 37°C. A standard session lasted for 25 min.

### Data Analyses

First, we bandpass filtered (1–50 Hz) and normalized (between 0 and 1) the raw EEG from each recording session. Then, the signal was binned using 5-s epochs, and the maximum peak-to-peak amplitude within each epoch was measured. The temperature was averaged within each time epoch and round to the nearest whole number (epochs with temperatures below 37°C or above 42°C were discarded). For each temperature in the analyzed range (37–42°C), we calculated the mean and SD of the maximum amplitude across epochs. Finally, we calculated the mean and SD of the maximum amplitude across recording sessions (*n* = 19). For statistical analyses, we ran binary comparisons with the non-parametric permutation test, with 20,000 iterations and an alpha level set at 0.05, using the MATLAB function permutation Test.m (Krol, 2021). This procedure allows testing the null hypothesis that two different groups come from the same distribution without requiring any assumption about the sampling distribution (Maris and Oostenveld, 2007; Cambiaghi et al., 2016).

### Frequency Domain

We divided each recording session in 5-s epochs, and for each epoch, we analyzed the power spectrum (estimated with the MATLAB function pspectrum) in four frequency bands separately: delta (1–4 Hz), theta (4–8 Hz), alpha (8–12 Hz), and beta (12–30 Hz). Then, we normalized the power spectra between 0 and 1 and calculated the average of the normalized power spectrum across epochs with the same temperature. To compare the results obtained at 37 and 42°C, we used the permutation test (20,000 iterations).

## Results

**FIGURE 2:**
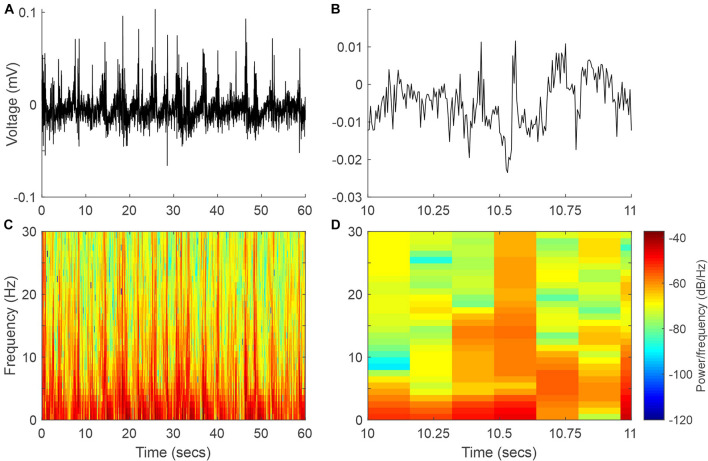
Example EEG signal and spectrogram at 37°C. **(A)** Sixty-second segment of the EEG signal. **(B)** One-second recording expanded from panel **(A)**. **(C,D)** Spectrograms of the EEG signals in panels **(A,B)**, respectively.

**FIGURE 3:**
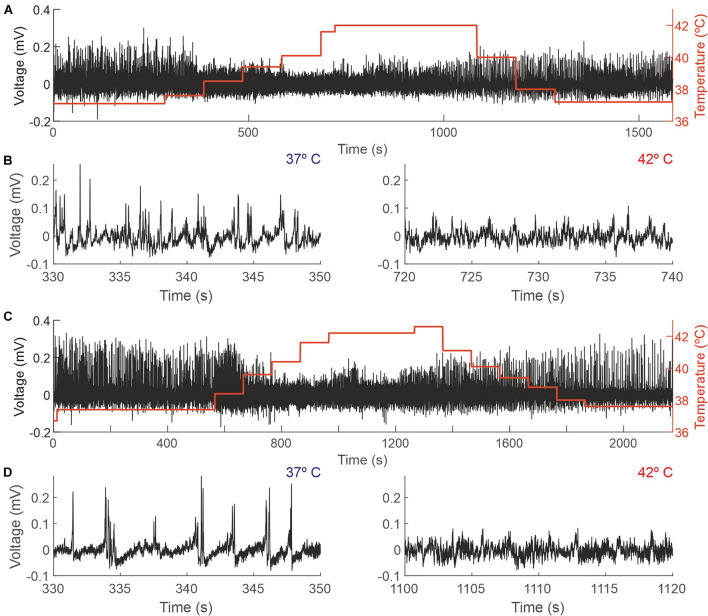
Changes in the EEG as a function of the core temperature recorded from two different mice. **(A,C)** Raw EEG data (black trace, left y-axis) and core temperature (red trace, right y-axis). **(B,D)** Details from the recordings depicted in panels **(A,C)**, respectively, showing 20 s during which the temperature of the mouse was 37°C (left panels) and 42°C (right panels).

Wild-type mouse cortical activity was continuously monitored by chronically implanted intracranial electrodes in the somatosensory (active) and visual cortices (reference). Animals were maintained in a state of light/medium anesthesia with sevoflurane. At 37°C, the EEG signal was dominated by low-frequency high-amplitude signals (Figures 2, 3). This kind of recording was maintained stable for long periods of time and was easily reproducible on different days in the same animal and even in different animals (Figure 3), hence representing a solid baseline for establishing comparisons. Figure 2 shows a representative 60-s EEG trace (A) and its spectrogram (C), where the dominance of lower frequencies is clearly visible. This figure also shows an expanded 1-s segment (B) and its spectrogram (D).

Increasing animal temperature had a dramatic effect on the recording amplitude, producing a reduction of 4.36% (±14.45) from 37 to 38°C and of 24.33% (±19.05) from 37 to 42°C. Figure 3 shows a representative example of a complete session in two different animals (panels A and C) and an expanded sample at 37 and 42 degrees, where those changes in amplitude are visible (panels B and D).

**FIGURE 4:**
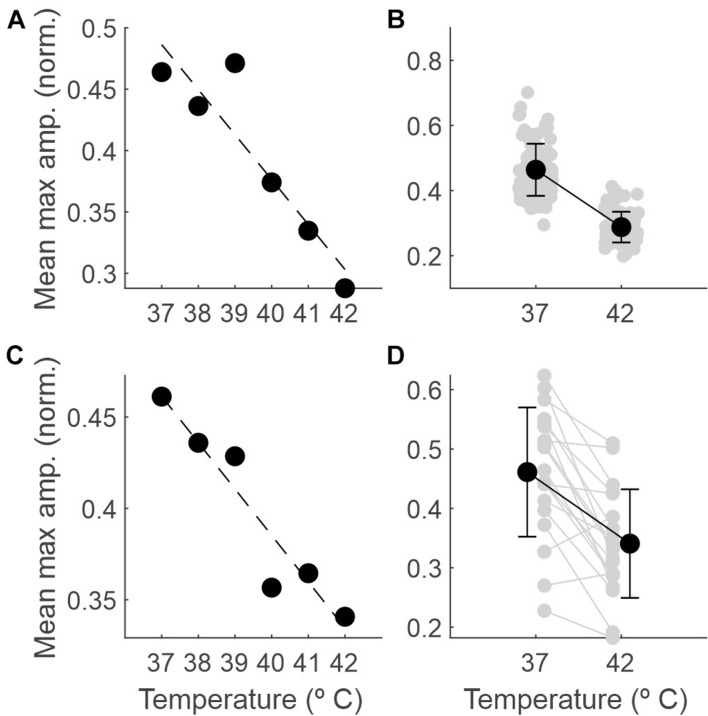
The amplitude of the EEG decreases as the temperature increases. **(A)** Maximum amplitude of the normalized EEG signal within each 5-s epoch (averaged across epochs) as a function of core temperature, for one example recording session. **(B)** Mean and SD (black dots) and individual values for each 5-s epoch (gray dots) comparing the maximum amplitude between 37 and 42°C for the same session. **(C)** The same as in panel **(A)**, but averaged across sessions (*n* = 19). **(D)** The same as in panel **(B)** but averaged across recording sessions (gray dots for individual sessions, *n* = 19).

Figure 4 shows how the EEG amplitude changes with temperature. In panel A, data from a single session illustrate a linear regression of averaged recorded amplitude (y = −0.037x + 1.842; R2 = 0.84). In panel C, the same data were averaged across all sessions (*n* = 19; y = −0.025x + 1.4; R2 = 0.9). Panels B and D show the comparison between the amplitudes at 37 and 42°C for one session and averaged across sessions, respectively. For the example session, the mean ± SD normalized amplitudes were 0.46 ± 0.08 and 0.29 ± 0.05 at 37 and 42°C, respectively; the mean ± SD amplitudes across sessions were 0.46 ± 0.11 and 0.34 ± 0.09, respectively. In both cases, the difference was significant (*p* < 0.0001 and *p* < 0.001, respectively; permutation tests).

In addition to the comparison of the extremes, we ran binary comparisons for the amplitude at other temperatures against that at 37°C and found significant differences from 40°C on (Table 1).

**TABLE 1:** Electroencephalography (EEG) amplitude averaged across recording sessions as a function of the temperature and *p*-values obtained by comparing the amplitude at each temperature against that at 37°C.

Temperature (°C)	Mean	SD	*p* (vs. 37°C)
37	0.461	0.109	–
38	0.436	0.122	0.504
39	0.429	0.118	0.385
40	0.357	0.131	**<0.05**
41	0.365	0.140	**<0.05**
42	0.341	0.091	**<0.001**

*Statistically significant values are marked in bold.*

### Frequency Analysis

The EEG recorded was characterized mainly by the different oscillations included in the signal; thus, we analyzed how body temperature modified those frequencies. In anesthetized animals, EEG is dominated by lower frequencies, but different components up to the beta range (12–30 Hz) are detectable (Figure 2B).

**FIGURE 5:**
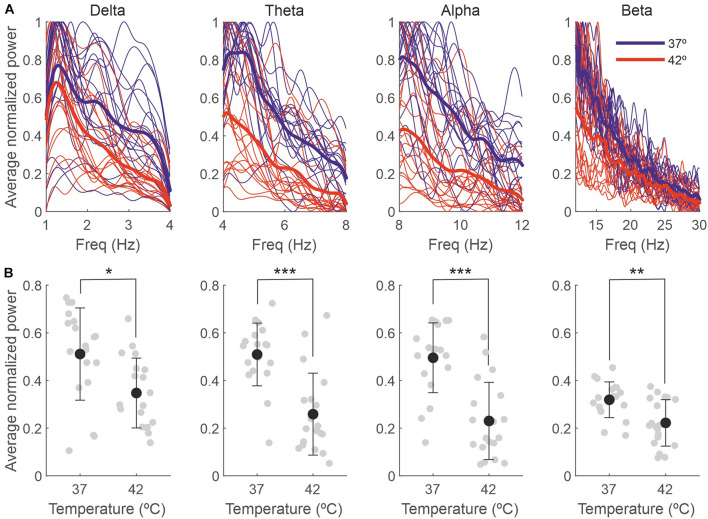
The effect of the temperature on the EEG is due to changes in the delta, theta, alpha and beta bands. **(A)** Power spectra for different frequency bands for each individual recording session, normalized between 0 and 1 and averaged across 5-s bins (thin lines) and averaged across sessions (thick lines). **(B)** Power averaged across frequencies of the spectra in panel **(A)**. Gray dots represent individual sessions (*n* = 19), and black dots represent the average across sessions (mean ± SD). *p < 0.01; **p < 0.005; ***p < 0.0001.

A temperature increase resulted in a reduction in the delta, theta, alpha and beta bands, as presented in Table 2 and Figure 5. For each frequency band, we calculated the mean normalized power for each session and temperature and compared 37 with 42°C. The averaged power for the different frequency bands is shown in panel B.

**TABLE 2:** Average of the normalized power for different frequency bands as a function of the temperature and *p*-values obtained by comparing 37°C with 42°C with a permutation test (*n* = 20,000 iterations).

	37°C		42°C		
	Mean	SD	Mean	SD	*p*
Delta	0.511	0.194	0.347	0.147	**<0.01**
Theta	0.509	0.132	0.259	0.172	**<0.0001**
Alpha	0.495	0.147	0.230	0.162	**<0.0001**
Beta	0.319	0.075	0.222	0.098	**<0.005**

*Statistically significant values are marked in bold.*

## Discussion

For many years, the effect of a temperature drop on EEG has been studied, mainly because of its clinical use, as it has protective effects on neurons in different conditions, such as acute ischemic stroke, traumatic brain injury, or inflammation of brain tissue (Sakoh and Gjedde, 2003). Conversely, hyperthermia is a more common condition in nature, but it has received less attention, at least taking into consideration its relationship with brain activity in healthy subjects. With this investigation, we aim to study the impact of hyperthermia on neural activity, a condition in which core temperature rises above the physiological threshold necessary to maintain normal body functions. Our results demonstrate that hyperthermia has an important impact on neural activity measured by EEG. Hyperthermia decreased not only the amplitude of the recorded signal but also induced a reduction in the delta, theta, alpha, and beta frequency bands when the temperature reached 42°C.

EEG signals represent a reliable tool for evaluating cortical function, a dynamic process that changes continuously and is affected by pathological conditions (e.g., infections) and physiological homeostasis. Regarding physiology, it is known that body temperature is an important factor driving neuronal activity, as the metabolism and integrity of brain cells depend on it (Mrozek et al., 2012).

EEG during febrile status has been intensively studied and analyzed and is usually related to other pathological processes, such as seizures, infections, cancer treatment (Barlogie et al., 1979; Reilly et al., 1980), or syphilis (Lin et al., 1953). However, the effect of hyperthermia on EEG in healthy subjects has not been deeply studied, even when fever is a common process that all of us suffer from at some point in life.

In the situations mentioned above, when EEG alterations are detected, it is often very difficult to disentangle which part can be attributed to the underlying pathological process and which to the temperature itself. Furthermore, the available results are not clear. Lifshitz et al. (1987) concluded after studying the EEG of patients without neurological pathologies during episodic fever that “fever in adults seems not to provoke rapidly reversible EEG changes.” This conclusion is completely different from the profound depression of recorded activity reported by Cabral et al. (1977). In both cases, the increase in temperature was a consequence of a previous condition that could affect the results. On the other hand, Reilly et al. (1980), studying cancer patients on hyperthermia treatment, found reversible changes in EEG signals related to temperatures above 41.5°C, mainly a decrease in the voltage signal and a shift to lower EEG frequencies.

The present study demonstrates a gradual reduction in signal intensity as the temperature increases, but in no case was there a suppression (Cabral et al., 1977) or an increase (Lin et al., 1953) of EEG signal as previously reported, indicating that those extreme results could be the consequence of the specific pathology. On the other hand, a gradual increase from 39 to 41°C followed by an abrupt decrease in EEG amplitude has also been reported in curarized rats (Ten Cate et al., 1949). Although it is a different animal model, our data fit with those last results.

Our results also showed that in baseline conditions (rectal temperature at 37°C), the EEG was dominated by low frequencies; as a consequence of anesthesia, delta frequencies rule the spectrum (Hagihira, 2015), and this predominance was maintained without changes even during the high temperature peaks. Although we observed significant decreases in all the frequency bands we analyzed, these differences where higher for the theta and alpha bands, suggesting they are more sensitive to high temperatures.

We must keep in mind that our control situation is anesthetized mice; hence, anesthesia influences both the initial EEG and the EEG obtained during hyperthermia. There are a number of studies describing the effect of different anesthetics on EEG signals (Brown et al., 2011; Akeju et al., 2014; Purdon et al., 2015) and showing that anesthesia tends to produce a common pattern on EEG, reducing the variability that characterizes the awake resting state. This pattern is characterized by a prominent presence of delta frequencies, usually with no differences between anesthetic agents. Delta frequencies have been proposed to be the default activity pattern of cortical networks since they have been detected in disconnected preparations (Sanchez-Vives and Mattia, 2014; Sanchez-Vives et al., 2017). Theta, alpha and low beta frequencies are also present in anesthetized animals, but the intensity and particular characteristics can vary with the anesthesia level and the anesthetic agent (Purdon et al., 2015). We are aware that our control situation cannot be defined as a normal physiological state, but on the other hand, it allows us to obtain a more stable standard initial point, decreasing the variability between animals. Additionally, anesthesia guarantees that changes are generalized, and two electrodes at distant places are sufficient to obtain a representative measurement of brain activity (Freye, 1990). We observed a reduction in delta, alpha, theta and beta frequencies, compatible with the described disruption of functional connectivity in the brain network under hyperthermia (Sun et al., 2013). It is known that body core temperature does not vary in the same way as brain temperature does (Kiyatkin, 2007). Therefore, we cannot establish a straightforward relationship between our measurements and real brain temperature at the same time, which is an experimental limitation. The fact that the experiments were conducted under anesthesia is another limitation, but this type of experiment, although possible, it would have been difficult to perform in awake animals, not only because of technical problems, but also because of ethical considerations. However, we do believe that whatever that relationship is, does not invalidate our observations.

These results show that hyperthermia is able to change cortical circuitry activity, affecting brain oscillations in anesthetized mice. Since alpha, theta and beta frequencies have been associated with different levels of awareness and different cognitive tasks (Wang, 2010; Fransen et al., 2016; Lundqvist et al., 2016) and hyperthermia has been negatively correlated with cognitive functions (Hancock and Vasmatzidis, 2003; Racinais et al., 2008; Gaoua et al., 2011), it is tentative to speculate that those changes in cortico-cortical synchronization could be behind some of the cognitive effects. Additionally, such effects open the door to the possibility that small temperature changes in awake animals may have a strong effect on cortical oscillations, affecting gamma rhythm (not detectable in our anesthetized animals), which is related to sensory stimulation, attention and cognitive tasks (Fries, 2009; Jia and Kohn, 2011; Cannon et al., 2014). Another open question worth considering in the future is whether longer periods of hyperthermia could produce stronger changes lasting longer or perhaps trigger some compensatory plastic phenomena. Finally, we should keep in mind that we measured rectal, not brain temperature, and there are a bunch of literature showing the differential responses.

In summary, acute elevation of rectal temperature is an important factor that is able to modify the electrical activity of the brain, probably impacting its metabolic activity. Our results showing the effect of hyperthermia on the amplitude and the synchronization of EEG signals pave the way for future research and represent a reference point for experiments involving a specific pathological condition in which temperature is a trigger (e.g., a mouse model of seizures where temperature is the trigger parameter for seizures).

## Data Availability Statement

The raw data supporting the conclusions of this article will be made available by the authors, without undue reservation.

## Ethics Statement

The animal study was reviewed and approved by the Committee for Animal Research of the University Hospital of A Coruña.

## Author Contributions

CL, CR, and JC designed the experiments and wrote the manuscript. CL and CR performed the experiments. JP-V performed the analysis. All authors contributed to the discussion and approved the final version of the manuscript.

## Conflict of Interest

The authors declare that the research was conducted in the absence of any commercial or financial relationships that could be construed as a potential conflict of interest.

## Publisher’s Note

All claims expressed in this article are solely those of the authors and do not necessarily represent those of their affiliated organizations, or those of the publisher, the editors and the reviewers. Any product that may be evaluated in this article, or claim that may be made by its manufacturer, is not guaranteed or endorsed by the publisher.
